# Integrative Cancer Care in a US Academic Cancer Centre: The Memorial Sloan–Kettering Experience

**DOI:** 10.3747/co.v15i0.276

**Published:** 2008-08

**Authors:** G. Deng

**Affiliations:** Memorial Sloan–Kettering Cancer Center, New York, NY, U.S.A

**Keywords:** Integrative medicine, complementary and alternative medicine, cancer, oncology

## Abstract

Various surveys show that interest in complementary and alternative medicine (cam) is high among cancer patients. Patients want to explore all options that may help their treatment. Many cam modalities offer patients an active role in their self-care, and the resulting sense of empowerment is very appealing. On the other hand, many unscrupulous marketeers promote alternative cancer “cures,” targeting cancer patients who are particularly vulnerable. Some alternative therapies can hurt patients by delaying effective treatment or by causing adverse effects or detrimental interactions with other medications. It is not in the best interest of cancer patients if they cannot get appropriate guidance on the use of cam from the health care professionals who are part of their cancer care team.

The Integrative Medicine Service at Memorial Sloan–Kettering Cancer Center in New York was established in 1999 to address patient interest in cam, to incorporate helpful complementary therapies into each patient’s overall treatment management, to guide patients in avoiding harmful alternative therapies, and to develop prospective research to evaluate the efficacy of cam modalities.

